# Resting-State Functional Network Topology Alterations of the Occipital Lobe Associated With Attention Impairment in Isolated Rapid Eye Movement Behavior Disorder

**DOI:** 10.3389/fnagi.2022.844483

**Published:** 2022-04-01

**Authors:** Chaofan Geng, Shenghui Wang, Zhonglin Li, Pengfei Xu, Yingying Bai, Yao Zhou, Xinyu Zhang, Yongli Li, Jiewen Zhang, Hongju Zhang

**Affiliations:** ^1^Henan University People’s Hospital, Henan Provincial People’s Hospital, Zhengzhou, China; ^2^Department of Neurology, Zhengzhou University People’s Hospital, Henan Provincial People’s Hospital, Zhengzhou, China; ^3^Department of Radiology, Zhengzhou University People’s Hospital, Zhengzhou, China; ^4^Department of Neurology, Henan Provincial People’s Hospital Affiliated to Xinxiang Medical University, Zhengzhou, China; ^5^Department of Functional Imaging, Henan Key Laboratory for Medical Imaging of Neurological Diseases, Zhengzhou, China

**Keywords:** idiopathic rapid eye movement sleep behavior disorder, resting-state functional magnetic resonance imaging, graph theory, network topology, functional disorders

## Abstract

**Purpose:**

This study investigates the topological properties of brain functional networks in patients with isolated rapid eye movement sleep behavior disorder (iRBD).

**Participants and Methods:**

A total of 21 patients with iRBD (iRBD group) and 22 healthy controls (HCs) were evaluated using resting-state functional MRI (rs-fMRI) and neuropsychological measures in cognitive and motor function. Data from rs-fMRI were analyzed using graph theory, which included small-world properties, network efficiency, network local efficiency, nodal shortest path, node efficiency, and network connectivity, as well as the relationship between behavioral characteristics and altered brain topological features.

**Results:**

Rey-Osterrieth complex figure test (ROCFT-copy), symbol digital modalities test (SDMT), auditory verbal learning test (AVLT)-N1, AVLT-N2, AVLT-N3, and AVLT-N1-3 scores were significantly lower in patients with iRBD than in HC (*P* < 0.05), while trail making test A (TMT-A), TMT-B, and Unified Parkinson’s Disease Rating Scale Part-III (UPDRS-III) scores were higher in patients with iRBD (*P* < 0.05). Compared with the HCs, patients with iRBD had no difference in the small-world attributes *(P* > 0.05). However, there was a significant decrease in network global efficiency (*P* = 0.0052) and network local efficiency (*P* = 0.0146), while an increase in characteristic path length (*P* = 0.0071). There was lower nodal efficiency in occipital gyrus and nodal shortest path in frontal, parietal, temporal lobe, and cingulate gyrus. Functional connectivities were decreased between the nodes of occipital with the regions where they had declined nodal shortest path. There was a positive correlation between TMT-A scores and the nodal efficiency of the right middle occipital gyrus (*R* = 0.602, *P* = 0.014).

**Conclusion:**

These results suggest that abnormal behaviors may be associated with disrupted brain network topology and functional connectivity in patients with iRBD and also provide novel insights to understand pathophysiological mechanisms in iRBD.

## Introduction

Isolated rapid eye movement (REM) sleep behavior disorder (iRBD) is characterized by the loss of normal muscular atonia during REM sleep (Sun et al., 2020). The prevalence is estimated to be 0.5–2% (St Louis and Boeve, 2017). Longitudinal cohort studies have shown that more than 80% of clinically diagnosed patients with iRBD develop neurogenerative disorders within 10 years, particularly α-synucleinopathies, including Parkinson’s disease (PD), dementia with Lewy bodies (DLB), and multiple system atrophy (Schenck et al., 2013; Postuma et al., 2015; Sun et al., 2020). Currently, iRBD is considered to be the prodromal stage of α-synucleinopathy and becomes the most important clinical symptom for predicting neurodegenerative diseases (Iranzo et al., 2017). Growing evidence shows that the cognitive impairment is more pronounced after conversion of iRBD to α-synucleinopathy (St Louis and Boeve, 2017). Therefore, exploring the characteristic pathophysiological mechanisms of iRBD has become a focus of research in order to halt or delay disease progression in its earliest stages.

Resting-state functional MRI (rs-fMRI) can indirectly respond to neuronal activity in brain regions through blood oxygen level-dependent (BOLD) (Ehgoetz Martens et al., 2020), which is widely used in neuropsychiatric disorders, including PD (Nagano-Saito et al., 2019), Alzheimer’s disease (Habib et al., 2017), and depression (Drysdale et al., 2017). Different methods have been used to explore disruptions of brain activity in iRBD using rs-fMRI, including regional homogeneity (ReHo), amplitude of low-frequency fluctuations (ALFF), functional connectivity (FC) (Campabadal et al., 2021), and so on. At present, rs-fMRI studies show decreased ReHo of the putamen, which is significantly associated with decreased dopamine transporter function in both iRBD and PD (Li G. et al., 2020), decreased functional connectivity between substantia nigra (SN) and striatum (Ellmore et al., 2013), and decreased functional connectivity among caudate nucleus, putamen, and pallidum (Rolinski et al., 2016). The brain has both integrative and dissociative functions for the processing of information (Li et al., 2018). Graph theory analysis has provided powerful tools to describe this complex system, using brain regions as nodes and interconnections between brain regions as edges (Bullmore and Bassett, 2011; Suo et al., 2015). Human brain networks have “small-world” properties, which can transmit information at a low cost and with the highest efficiency. One of the few studies that have shown significant alterations in structural connectivity compared with healthy controls (HCs) (Park et al., 2019). Another study has reported decreased node centrality located in the left superior parietal lobule, which was related to cognitive impairment in patients with iRBD who were taking benzodiazepines (Campabadal et al., 2020). However, it is not known whether the change was related to medication.

Therefore, the aim of this study was to explore the differences in brain network topological properties between drug-naive iRBD and HCs using graph theory analysis. It is helpful to understand the pathological basis of cognitive impairment in iRBD and provide pathological evidence for converted iRBD.

## Materials and Methods

### Participants

A total of 21 patients with iRBD who were first diagnosed and did not receive treatment were recruited in the outpatient from the neurology department of Henan Provincial People’s Hospital. The International Classification of Sleep Disorder (ICSD-3) diagnostic criteria for RBD were used to screen patients with iRBD, which was also later confirmed by polysomnography (PSG) (Sateia, 2014). Twenty-two age-, gender-, and education-matched outpatient health screeners were selected as the HC group during the same period.

Exclusion criteria for iRBD were as follows: (1) cognitive impairment [with the Mini-Mental State Examination (MMSE) < 26 scores (Gagnon et al., 2010)]; (2) other neurological disorders, such as Lewy body dementia (LBD) and Parkinson’s syndrome; and (3) patients with severe cardiac, pulmonary, hepatic, renal, and endocrine systemic diseases, and malignant tumors; history of psychiatric diseases, such as anxiety and depression; patients with secondary RBD; other sleep disorders; patients with contraindications to magnetic resonance examination; exclude taking medications including antipsychotics, electroconvulsive therapy (ECT), and alcohol-dependent patients; exclude illiterate, deaf, and other uncooperative patients.

The exclusion criteria of HCs were as follows: those who were contraindicated for MRI examinations; history of neuropsychiatric diseases and brain trauma; other sleep disorders; and those who had a history of long-term alcohol abuse or other drug abuse.

The research protocol of this study was approved by the Research Ethics Committee of Henan Provincial People’s Hospital and all patients signed written informed consent (No. 201705).

### Neuropsychological and Clinical Assessment

All participants underwent routine assessments, including standardized neuropsychological and clinical assessment by a trained doctor in an evaluator-blinder fashion.

The Hoehn-Yahr (H-Y) stage and the Unified Parkinson’s Disease Rating Scale Part-III (UPDRS-III) were used to assess motor function in all subjects.

The MMSE was used for global cognitive screening (Gagnon et al., 2010). The digit ordering test-A (DOT-A) (Werheid et al., 2002) and trail making test A (TMT-A) (Balzano et al., 2006) were used to assess processing attention. Executive function was observed using TMT-B (Zhao et al., 2013) and symbol digital modalities test (SDMT) (Balzano et al., 2006), and visuospatial ability was assessed using Rey-Osterrieth complex figure test (ROCFT-copy) (Shin et al., 2006). Verbal memory was evaluated using the auditory verbal learning test (AVLT) (Guo et al., 2009). AVLT-N1, N2, and N3 were for immediate recall during the learning phase, which is closely related to attention/working memory (Zhang et al., 2021).

### Magnetic Resonance Images Acquisition and Preprocessing

Magnetic resonance images were acquired at the Medical Imaging Center of Henan Provincial People’s Hospital using a Discovery 750 3.0 T MRI scanner from GE, United States, with an 8-channel phased-array head-neck coil, and the head was fixed with foam pads during scanning. All subjects were instructed to keep their eyes closed, not deliberately think about things and try to remain the head fixed. The scan sequence and parameters are as follows: (1) to exclude abnormalities in brain structures, using a 3D fast spoiled gradient recalled (FSPGR) sequence with the following scanning parameters: repetition time (TR) = 8.2 ms, echo time (TE) = 3.22 ms, inversion time (TI) = 450 ms, matrix size = 256 × 256, slice thickness = 1.0 mm, field of view (FOV) = 240 mm, flip angle (FA) = 12°, and 156 layers that were scanned. (2) A standard weighted gradient-echo planar imaging (GRE-EPI) sequence was used to perform the resting-state functional scan using the BOLD technique with the following scanning parameters: TR = 2,000 ms, TE = 30 ms, TI = 450 ms, FOV = 240 mm, matrix size = 64 × 64, slice thickness = 4.0 mm, flip angle (FA) = 90°, and a total of 39 layers were scanned.

Data preprocessing of images was performed using the statistical parametric mapping software DPARSF.^1^ The first 10 scans of the rs-fMRI images were discarded to reach magnetization equilibrium; the remaining fMRI data were time-corrected and head-motion corrected to exclude the subjects with maximum head movement translation > 3 mm or rotation angle > 3°; (Campabadal et al., 2020) the images were spatially normalized to the MNI and resampled to 3-mm cubic voxels; the datasets were spatially smoothed using a 4-mm full-width at half-maximum (FWHM) Gaussian spatial kernel; the images were detrended and filtered at 0.01–0.08 Hz to exclude the effect of physiological noise. The interference of head motion, whole-brain signal, white matter signal, and cerebrospinal fluid signal was eliminated using the covariate regression method to finalize the rs-fMRI image data preprocessing.

### Network Construction and Graph Theory Analysis

To construct a functional connectivity brain network, this study used automated anatomical labeling (ALL) mapping to delineate brain regions, dividing each subject’s brain into 90 cortical and subcortical regions of interest (Tzourio-Mazoyer et al., 2002), with each brain region considered as a network node. To determine the edges of the network, the average time series of each region was calculated. The Pearson’s correlation coefficients between the mean time series of all possible pairs of the 90 brain regions were calculated as the edges of the network. Then, Fisher’s *r*-to-*z* transformation was used to improve data distributions for parametric statistical analysis and derive a 90 × 90 correlation weight matrix (Guan et al., 2019).

The topological characteristics of the brain functional network were obtained by graph theory using GRETNA^2^ software in MATLAB environment. This study uses network sparsity (S) as a threshold metric. S is defined as the ratio of the number of edges actually present in the network to the number of maximum possible edges after setting a fixed threshold (Huang et al., 2019). The choice of S as the threshold metric ensures that each threshold network has the same number of nodes and edges, thus minimizing the difference in the overall correlation strength between groups. We used a wide range of threshold levels in this study (0.05 < S < 0.50, interval 0.05), calculating the area under the curve (AUC) for each network threshold metric, which provides a summary scalar for characterizing brain network topology independently of a single threshold selection, for a more sensitive study of group differences in these networks.

At each threshold, the topological properties of the brain functional network are calculated at two levels, global and local, respectively. There are two types of global metrics, namely, small-world parameters (Watts and Strogatz, 1998), including clustering coefficient (*C*_*p*_), characteristic path length (*L*_*p*_), normalized clustering coefficient (γ), normalized characteristic path length (λ), and scalar small-world (σ), and network efficiency (Berdoz et al., 2001), including global efficiency *E*_*glob*_ and local efficiency *E*_*loc*_. Small-world properties, σ = γ/λ, σ > 1; γ > 1 and λ≈ 1, consistent with graph theory, are required for efficient integration and separation function (Huang et al., 2019; Yang et al., 2019). Nodal metrics of functional networks include degree centrality, nodal efficiency, and nodal shortest path (Guan et al., 2019). It is worth noting that we calculated the average AUC for the above network parameters at different threshold metrics for intergroup comparison.

### Statistical Analysis

The SPSS 19.0 statistical software was used for the analysis of clinical data in the iRBD and HC groups. The normality of the distribution was tested using the Kolmogorov–Smirnov test. Normally distributed measures were expressed as mean ± SD and compared between the two groups using the *t*-test. Non-normally distributed measures were expressed as medians and quartiles [M (P25, P75)] and compared between the two groups using the Mann-Whitney *U*-test. Count data were expressed as relative number composition ratio (%) or rate (%), and Fisher’s exact probability analysis was used. Differences were considered statistically significant at *P* < 0.05. Brain topography data from the iRBD and HC groups were compared using a two-sample *t*-test with a statistical threshold of *P* < 0.05 using GRETNA software and corrected for statistical results by false discovery rate (FDR). *P* < 0.05 was set as a statistically significant difference. Partial correlation analysis of differential iRBD node matrices with their abnormal clinical scores was performed using SPSS 19.0 (to remove the effect of gender, age, and education covariates), and *P* < 0.05 was considered a statistically significant difference. Images were generated using BrainNet Viewer.^3^

## Results

### Demographic and Clinical Characteristics

No significant differences were found in the age, gender, education, MMSE, H-Y scale, apnea-hypopnea index (AHI), and periodic limb movement index (PLMI) between the iRBD group and HC group (*P* > 0.05, Table 1). Compared with HC, the iRBD showed a significant decrease in ROCFT-copy, SDMT, AVLT-N1, AVLT-N2, AVLT-N3, and AVLTN1-3 and an increase in TMT-A, TMT-B, and UPDRSIII (*P* < 0.05, Table 1).

**TABLE 1:** Demographic and clinical characteristics of the subjects.

Characteristics	iRBD (*n* = 21)	HC (*n* = 22)	*T*/*U*-test value	*P*-value
Age (Years)	61.00 ± 10.68	60.27 ± 7.60	0.26*^a^*	0.779
Level of education (Years)	8.81 ± 3.40	8.86 ± 3.14	−0.05*^a^*	0.957
Male	14	9	2.865*^b^*	0.091
MMSE	27 (26, 28)	28 (27, 28)	−0.76	0.448
ROCFT-copy M (P25, P75), score	32.00 (31.00, 34.00)	34.50 (32.00, 36.00)	−2.01*^c^*	**0.045**
AVLT-N1 mean ± SD, score	3.86 ± 1.82	5.00 ± 1.23	−2.42*^a^*	**0.020**
AVLT-N2 mean ± SD, score	5.29 ± 1.86	6.64 ± 1.14	−2.873*^a^*	**0.006**
AVLT-N3 M (P25, P75), score	6.00 (5.00, 8.00)	8.00 (7.00, 9.00)	−2.38*^c^*	**0.017**
AVLTN1-3 mean ± SD, score	15.57 ± 5.33	19.32 ± 3.55	−2.73	**0.009**
SDMT M (P25, P75), score	23.00 (21.50, 31.00)	33.00 (30.00, 36.00)	−3.36*^c^*	**0.001**
TMT-A M (P25, P75), score	88 (70, 120)	60 (49, 80.5)	−2.644*^c^*	**0.008**
TMT-B M (P25, P75), score	194 (180, 210)	112 (100, 150)	−3.008*^c^*	**0.003**
DOT M (P25, P75), score	5 (4, 5)	6 (5, 6)	−1.762*^c^*	0.078
UPDRSIII (points)	0 (0, 2)	0 (0, 1)	−3.613	**0.000**
H-Y scale (grades)	0 (0, 0)	0 (0, 0)	187.000*^c^*	0.063

*iRBD, isolated rapid eye movement sleep behavior disorder; HCs, healthy controls. MMSE, Mini-Mental State Examination; ROCFT, Rey-Osterrieth complex figure test; AVLT, auditory verbal learning test; SDMT, symbol digit modalities test; TMT, trail making test; DOT, digit ordering test; MDS-UPDRSIII, movement disorder society unified Parkinson’s disease rating scale motor section; AHI, apnea-hypopnea index; PLMI, periodic limb movement index. Data are presented as mean ± SD, or median (interquartile range) as appropriate. For the comparisons of cognitive tests, P < 0.05 is shown in bold.*

*^a^Student’s t-test for two groups.*

*^b^Chi-square test for two groups.*

*^c^Mann-Whitney U-test for two groups.*

### Global Topological Organization of the Functional Connectome

**FIGURE 1:**
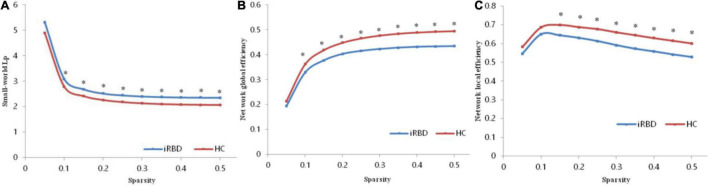
Altered global topological parameters of the functional connectome across different sparsity thresholds (0.05–0.50). **(A)** Small-world Lp of the functional connectome. **(B)** Network global efficiency of the functional connectome. **(C)** Network global efficiency of the functional connectome. Black stars indicate where the difference between patients with iRBD and HCs was significant (*P* < 0.05). iRBD, isolated rapid eye movement sleep behavior disorder; HC, healthy control.

Both patients with iRBD and HCs showed small-world topology in the brain functional connectome, as depicted by the high *C*_*p*_ (γ > 1) and similar *L*_*p*_ (λ≈ 1) compared with matched random networks, which can be unified by a scalar measure σ (σ > 1) (Humphries et al., 2006). However, no significant differences were found in topological properties of small-world except for *L*_*p*_. Compared with the HC, the iRBD group showed a significantly increased *L*_*p*_ (*P* = 0.0071, Figure 1A) and a significantly decreased *E*_*glob*_ (*P* = 0.0052, Figure 1B) and *E*_*loc*_ *(P* = 0.0146, Figure 1C).

### Regional Topological Organization of the Functional Connectome

Compared with the HC group, the iRBD group showed decreased nodal efficiency in left lingual gyrus, right middle occipital gyrus, bilateral superior occipital gyrus, as well as decreased nodal short-path in left middle cingulum gyrus, supramarginal gyrus, central paracentral lobule, right inferior frontal triangular gyrus, superior motor area, rectus gyrus, Rolandic operculum, bilateral postcentral gyrus, precentral gyrus, and superior temporal gyrus (Table 2).

**TABLE 2:** Regions showing disrupted nodal characteristics in patients with RBD compared with HCs.

	Brain regions	*T*-value	*P*-value*/P_*cor*_* valu*e*
Nodal efficiency	LING.L	−3.7730	**0.0010/0.0006**
	SOG.L	−3.7381	**0.0010/0.0006**
	SOG.R	−3.9751	**0.0010/0.0003**
	MOG.R	−3.5647	**0.0010/0.0010**
Nodal shortest path	PreCG.L	−3.1964	**0.0072/0.0028**
	PreCG.R	−3.4879	**0.0072/0.0013**
	IFGtriang.R	−2.8603	**0.0072/0.0069**
	ROL.R	−3.5683	**0.0072/0.0010**
	SMA.R	−3.0854	**0.0072/0.0038**
	REC.R	−3.1071	**0.0072/0.0036**
	MCG.L	−3.5923	**0.0072/0.0009**
	PoCG.L	−2.8450	**0.0072/0.0072**
	PoCG.R	−3.2423	**0.0072/0.0025**
	SMG.L	−2.9888	**0.0072/0.0050**
	PCL.L	−2.8566	**0.0072/0.0070**
	STG.L	−3.2492	**0.0072/0.0025**
	STG.R	−3.8196	**0.0072/0.0005**

*P-value (P < 0.01, FDR-corrected). iRBD, isolated rapid eye movement sleep behavior disorder; HCs, healthy controls; MCG, middle cingulum gyrus; LING, lingual gyrus; SOG, superior occipital gyrus; MOG, middle occipital gyrus; PoCG, postcentral gyrus; SMG, supramarginal gyrus; IFG-triang, inferior frontal triangular gyrus; PCL, central paracentral lobule; SMA, superior motor area; PreCG, precentral gyrus; REC, rectus gyrus; ROL, Rolandic operculum; STG, superior temporal gyrus. Regions are considered abnormal in the iRBD patients if they exhibited significant between-group differences in at least one of the three nodal centralities (shown in bold font).*

### Isolated Rapid Eye Movement Sleep Behavior Disorder-Related Alterations in Functional Connectivity

**FIGURE 2:**
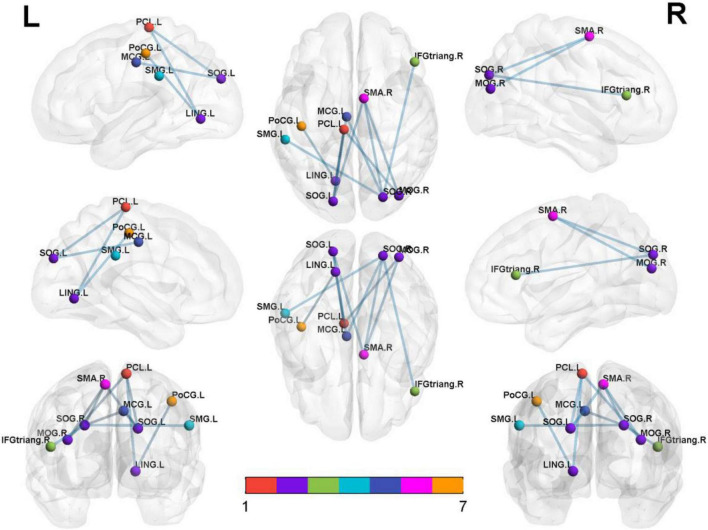
Significantly altered nodal centralities of the brain functional connectome in patients with iRBD, compared with HCs (*P* < 0.01, FDR-corrected). These connections formed a single connected network with 14 nodes and 27 connections (*P* < 0.05, corrected by NBS). To facilitate differentiation, different brain regions were indicated by different colors. Lines represent decreased functional connectivity strength in patients with iRBD. Details are listed in Table 3. MCG, middle cingulum gyrus; LING, lingual gyrus; SOG, superior occipital gyrus; MOG, middle occipital gyrus; PoCG, postcentral gyrus; SMG, supramarginal gyrus; IFG-triang, inferior frontal triangular gyrus; PCL, central paracentral lobule; SMA, superior motor area; Precg, precentral gyrus; REC, rectus gyrus; ROL, Rolandic operculum; STG, superior temporal gyrus.

Significantly decreased brain network was found in the iRBD group compared with the HC group [*P* < 0.05, corrected by network-based statistic (NBS)] (Zalesky et al., 2010). The brain network had 17 nodal including left middle cingulum gyrus, lingual gyrus, supramarginal gyrus, central paracentral lobule, right middle pccipital gyrus, inferior frontal triangular gyrus, superior motor area, rectus gyrus, Rolandic operculum, bilateral superior occipital gyrus, postcentral gyrus, precentral gyrus, and superior temporal gyrus. Compared with the HC group, the decreased connections appeared not only in right middle occipital gyrus with right superior motor area and left central paracentral lobule but also in left lingual gyrus with left central paracentral lobule and postcentral gyrus. In addition, decreased connections were seen in the left superior occipital gyrus with left lingual gyrus, central paracentral lobule, and right superior motor area. Apart from the above-mentioned, the decreased connections also happened in the right superior occipital gyrus with the left lingual gyrus, supramarginal gyrus, right superior motor area, and inferior frontal triangular gyrus. Nevertheless, no different connections were displayed between the iRBD group and HC group among the right middle occipital gyrus, left lingual gyrus, and bilateral superior occipital gyrus (Figure 2 and Table 3).

**TABLE 3:** Significantly decreased functional connectivities in patients with iRBD compared with HCs.

Brain regions	Brain regions	*T*-value	*P*-value*/P_*cor*_* value
MOG.R	SMA.R	−3.3285	**0.01/0.0020**
	PCL.L	−3.3378	**0.01/0.0019**
SOG.L	MCG.L	−3.5022	**0.01/0.0012**
	SMA.R	−3.8208	**0.01/0.0005**
	PCL.L	−3.7272	**0.01/0.0006**
SOG.R	MCG.L	−3.888	**0.01/0.0004**
	SMA.R	−3.7668	**0.01/0.0006**
	IFGtriang.R	−3.5815	**0.01/0.0010**
	SMG.L	−3.4823	**0.01/0.0013**
LING.L	PCL.L	−4.0851	**0.01/0.0002**
	PoCG.L	−3.5777	**0.01/0.0019**

*P-value (P < 0.01, FDR-corrected). iRBD, isolated rapid eye movement sleep behavior disorder; HCs, healthy controls; MCG, middle cingulum gyrus; LING, lingual gyrus; SOG, superior occipital gyrus; MOG, middle occipital gyrus; PoCG, postcentral gyrus; SMG, supramarginal gyrus; IFG-triang, inferior frontal triangular gyrus; PCL, central paracentral lobule; SMA, superior motor area. Regions are considered abnormal in the iRBD patients if they exhibited significant between-group differences in at least one of the three nodal centralities (shown in bold font).*

### Relationships Between Network Metrics and Clinical Variables

**FIGURE 3:**
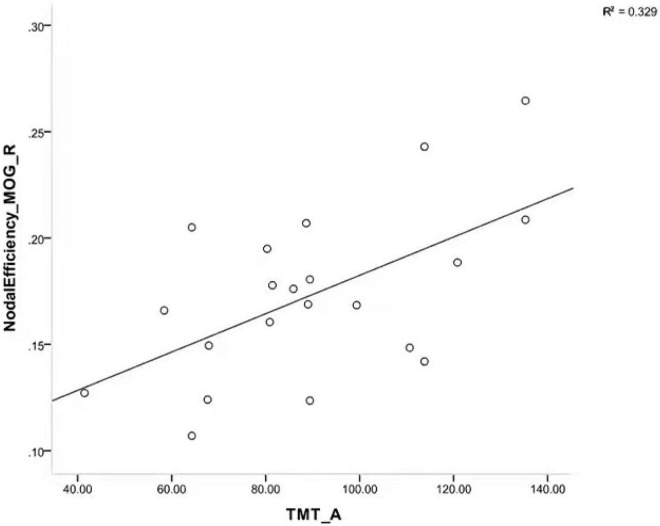
Scatterplot showing a significant positive correlation between TMT-A scores and node efficiency of right MOG in patients with iRBD. TMT-A, trail making test part A; MOG, middle occipital gyrus; iRBD, isolated rapid eye movement sleep behavior disorder.

We calculated the partial correlation between clinical characteristics and the global network metrics and regional nodal parameters, with age, gender, and education as covariates. The results showed that a significant positive correlation was only found in the TMT-A score with node efficiency of the right middle occipital gyrus (*R* = 0.602, *P* = 0.014; Figure 3). However, the rest of any clinical characteristics had no significant correlation with the other global network metrics and regional nodal parameters (*P* > 0.05).

## Discussion

In this study, we had three main findings. First, patients with iRBD presented with impairment in multiple cognitive domains and motor. Second, graph theory analysis revealed increased *L*_*p*_ and reduced *E*_*glob*_ and *Eloc* in iRBD. Additionally, decreased nodal efficiency in the occipital gyrus and decreased nodal shortest path involving multiple cortical regions were seen, whereas there appeared to be decreased FC between the occipital gyrus and other cortical regions. Third, TMT-A scores positively correlated with nodal efficiency of the right mid-occipital gyrus. All in all, the abnormal functional topological properties were associated with clinical characteristics in patients with iRBD.

### The Pathological Basis of Motor and Cognitive Impairment in Isolated Rapid Eye Movement Sleep Behavior Disorder

The iRBD is a non-motor symptom of PD and is considered the preclinical stage of PD. A previous study reported that patients with iRBD showed cognitive, memory, and motor deficits (Li et al., 2016), which was similar to our results.

Small-world networks have properties with high clustering coefficients and the shortest path lengths between regular and random networks and have relatively high local and global efficiency in information transfer and processing (Li et al., 2021). Human brain has small-world properties in information processing (Chen et al., 2017). Studies have found the altered small-world properties in many disorders, such as major depression (Zhang et al., 2011), PTSD (Suo et al., 2017), and schizophrenia (Wang et al., 2012). Our study showed that the small-world properties of patients with iRBD are not yet abnormal, implying that patients with iRBD are still in the early stages of the disease and still have a low energy consumption mode in terms of information processing. *E*_*glob*_ measures the global efficiency of parallel information transmission of the network; *L*_*p*_ reflects the information transmission capacity of the network nodes, which reflects the global transmission capacity. The smaller the value, the stronger the transmission capacity (Li et al., 2018). Our results showed decreased *E*_*glob*_ and increased *L*_*p*_ in patients with iRBD. *E*_*loc*_ is the average of the local efficiency of all nodes, which measures the local information transmission capacity of the network and reflects, to a certain extent, the network’s ability to defend against random attacks (Li et al., 2018). Our results showed that *E*_*loc*_ in patients with iRBD is significantly reduced, and their defensive capability is decreased. Previous studies have found that patients with early PD have reduced *E*_*glob*_ in structural brain networks (Li et al., 2018). Our results further confirm that iRBD is similar to PD and may be the preclinical stage of PD.

The nodal shortest path can reflect the ability to transmit information, which can save system resources and reduce the energy consumption of network information transmission. A decreased nodal shortest path indicates that the node is more efficient (Chen et al., 2017). Reduced nodal shortest paths were mainly located in the sensorimotor network (SMN) (He et al., 2021). Accelerated information transfer in SMN may be related to abnormal nocturnal motor in iRBD, or it could be a compensatory mechanism. The default mode network (DMN) is suppressed when awake and active when asleep (Marques et al., 2018) and controlled by the salience network (SN). Our study showed that reduced nodal shortest paths appeared in the supramarginal gyrus, which is one node of DMN, and the middle cingulate gyrus, which is part of SN. These changes may be associated with abnormal nighttime dreams. The superior temporal gyrus is a node that belongs to the auditory network (AUN), which is associated with emotion and memory (Takahashi et al., 2010). Decreased nodal shortest path of the superior temporal gyrus may be associated with dysfunction of emotion and memory in iRBD. The prefrontal triangle, insula, and rectus gyrus are associated with cognition, memory, thinking intuition, problem-solving skills, as well as emotion (Friedman and Robbins, 2022). Reduced nodal shortest paths in these regions disturbed information processing in iRBD, which may underlie the impairment of attention and executive. Our study showed that reduced nodal efficiency located in the left lingual gyrus, right middle occipital gyrus, and bilateral superior occipital gyrus belonging to the visual network (Goldman et al., 2014), and visuospatial deficits may be associated with it in iRBD.

### The Important Role of the Occipital Lobe in the Pathogenesis of Isolated Rapid Eye Movement Sleep Behavior Disorder

The occipital lobe may be more prone to early damage in patients with DLB. The occipital lobe is related to dreams (Schwarzkopf et al., 2011) and visual-spatial memories (Li K. et al., 2020). Our results illustrated that the information processing capacity of the occipital lobe was reduced in iRBD, and implied brain regions were damaged. Previous studies have shown that the occipital lobe of glucose metabolism decreased in patients with iRBD (Han et al., 2019) and spread to the parietal lobe following the development of DLB (Fujishiro et al., 2013), in contrast to PD in the frontal and parietal cortex (Hou et al., 2010). Patients with iRBD who had cognitive dysfunction had lower occipital gray matter volume (Rahayel et al., 2018) and lower strength of network connections in the posterior brain (Byun et al., 2020), indicating that changes in the occipital lobe appear in the early stages of the disease. Reduced nodal efficiency in the occipital may be associated with frequent dreams and visuospatial disturbances in patients with iRBD.

Previous studies have found that patients with iRBD had hyperperfusion in the superior motor area during episodes and (Mayer et al., 2015) volume reduction in the postcentral gyrus (Han et al., 2019). Our results also showed decreased functional connectivity appeared in the occipital lobe to the left middle cingulate gyrus, the right inferior frontal triangle gyrus, and the left superior marginal gyrus, those areas involved in the DMN, the SN, and the cognitive brain regions of the prefrontal lobe, and were affected by lower nodal efficiency of the occipital lobe. Previous studies have found that prefrontal cortex atrophy was related to attention and executive function in iRBD (Hou et al., 2010), and having hyperperfusion may be a compensatory response to atrophy increasing the risk of conversion from iRBD to α-synucleinopathy (Ye et al., 2020). Both the iRBD and the PD with RBD have shown the reduction in cingulate gyrus volume (Blanc et al., 2016), which was associated with frequent dreams in patients with PD (Radziunas et al., 2018). Reduced functional connectivities may be important for motor and cognitive impairment in iRBD. Functional changes often precede structural damage, so fMRI can help us detect pathological changes in the disease early. Further studies are needed to confirm this speculation. In addition, a growing number of recent studies suggest that the lateralization of motor symptoms in Parkinson’s disease (PD) is the result of asymmetric degeneration of the nigrostriatal pathway (Kurlawala et al., 2021), which may induce the altered unilateral connectivity of the right middle occipital gyrus. This study found that TMT-A scores were positively correlated with nodal efficiency in the right middle occipital gyrus, suggesting that attention is affected by the nodal efficiency of the right middle occipital gyrus in patients with iRBD. We think that occipital lobe damage may be associated with iRBD episodes of nocturnal dream symptoms, which manifest as vivid visual images, possibly as a compensatory mechanism. The change is the same as Iranzo’s results, which show abnormalities in the posterior brain functional connectivity in patients with iRBD (Campabadal et al., 2020). The middle occipital gyrus is a key brain region in the visual cognitive network, involved in facial recognition and emotional cognition, and plays an important role in visual information integration (Tu et al., 2013). Once damaged, it can lead not only to visual impairment but also to memory deficits and motor perception impairment (Thientunyakit et al., 2021). Therefore, in this study, the abnormal nodal efficiency of the middle occipital gyrus may have played a role in interfering with attention ability.

There are also some limitations in this study. First, the segmentation strategy and spatial scale of node definition have a great impact on network properties. Difference AAL template may lead to imparity in graph theory results. Second, PSG is important for objective quantification of REM sleep, and our study did not introduce the parameters of REM sleep in PSG into the analysis, and future studies combining PSG parameters with topological properties will help us to deeply understand the clinical symptoms and pathophysiological mechanisms in iRBD. Third, since this is a cross-sectional study, future longitudinal cohort studies are required to determine whether occipital topology abnormalities can predict the iRBD conversion. Fourth, physiological noise, including respiration, head movement, and cardiac fluctuations, may affect our results. Fifth, the sample size of the included studies is relatively small, and future studies expand the sample size and combine it with multicenter studies to further validate the results of this study.

## Conclusion

This study results indicate that the brain functional network topology alterations of the occipital lobe associated with attention impairment are in line with previous literature in patients with iRBD (Campabadal et al., 2020) and according to the characteristic pathological changes of DLB (Fujishiro et al., 2013). As an important node with decreased nodal efficiency, the occipital lobe can cause decreased FC involving SMN, DMN, SN, and prefrontal cognitive brain regions, which would be an important basis for nocturnal symptoms of iRBD and cognitive-motor impairment, and the regions are also involved in PD with dementia. So, iRBD will be widely heterogeneous in future conversions. This finding will help us to understand the pathophysiological mechanisms of iRBD.

## Data Availability Statement

The data are available via contacting the corresponding author. Requests to access these datasets should be directed to HZ, hongjuz@sina.com.

## Ethics Statement

The study was approved by the Ethics Committee of Henan Provincial People’s Hospital (No. 201705) and Henan Province Medical Science and Technology Tackling Provincial Ministry Key Projects (SBGJ202102033). In addition, all participants signed written informed consent prior to participation. This study was conducted in accordance with the Declaration of Helsinki.

## Author Contributions

HZ: conceptualization, resources, and supervision. CG: writing. SW, YB, and YZ: methodology. XZ, PX, ZL, YL, and HZ: data analysis. All authors contributed to this study and approved the submitted version.

## Conflict of Interest

The authors declare that the research was conducted in the absence of any commercial or financial relationships that could be construed as a potential conflict of interest.

## Publisher’s Note

All claims expressed in this article are solely those of the authors and do not necessarily represent those of their affiliated organizations, or those of the publisher, the editors and the reviewers. Any product that may be evaluated in this article, or claim that may be made by its manufacturer, is not guaranteed or endorsed by the publisher.

## Footnotes

1
http://www.restfmri.net/

2
http://www.nitrc.org/projects/gretna/

3
http://www.nitrc.org/projects/bnv/
